# Methamphetamine effects on blood-brain barrier structure and function

**DOI:** 10.3389/fnins.2015.00069

**Published:** 2015-03-04

**Authors:** Nicole A. Northrop, Bryan K. Yamamoto

**Affiliations:** Department of Neurosciences, University of Toledo College of MedicineToledo, OH, USA

**Keywords:** methamphetamine, blood-brain barrier, oxidative stress, glutamate, neuroinflammation, tight junction

## Abstract

Methamphetamine (Meth) is a widely abuse psychostimulant. Traditionally, studies have focused on the neurotoxic effects of Meth on monoaminergic neurotransmitter terminals. Recently, both *in vitro* and *in vivo* studies have investigated the effects of Meth on the BBB and found that Meth produces a decrease in BBB structural proteins and an increase in BBB permeability to various molecules. Moreover, preclinical studies are validated by clinical studies in which human Meth users have increased concentrations of toxins in the brain. Therefore, this review will focus on the structural and functional disruption of the BBB caused by Meth and the mechanisms that contribute to Meth-induced BBB disruption. The review will reveal that the mechanisms by which Meth damages dopamine and serotonin terminals are similar to the mechanisms by which the blood-brain barrier (BBB) is damaged. Furthermore, this review will cover the factors that are known to potentiate the effects of Meth (McCann et al., 1998) on the BBB, such as stress and HIV, both of which are co-morbid conditions associated with Meth abuse. Overall, the goal of this review is to demonstrate that the scope of damage produced by Meth goes beyond damage to monoaminergic neurotransmitter systems to include BBB disruption as well as provide a rationale for investigating therapeutics to treat Meth-induced BBB disruption. Since a breach of the BBB can have a multitude of consequences, therapies directed toward the treatment of BBB disruption may help to ameliorate the long-term neurodegeneration and cognitive deficits produced by Meth and possibly even Meth addiction.

## Introduction

Methamphetamine (Meth) is a widely abused psychostimulant that causes long-term monoamine neurotransmitter depletions. The well-established mechanisms responsible for Meth-induced damage to monoaminergic terminals are not selective but likely target other cell types and tissues as well. In this review, the focus will be on the effects of Meth and comorbid factors on blood-brain barrier (BBB) structure and function and how the mechanisms of Meth-induced monoaminergic damage can also contribute to BBB damage. In addition, potential consequences of Meth-induced BBB disruption and the importance of further research regarding Meth-induced BBB disruption will be discussed.

## Meth-induced neurotoxicity

Meth produces long-term neuronal damage in humans, non-human primates and rodents. Positron emission tomography (PET) and proton magnetic resonance spectroscopy (MRS) studies in abstinent human Meth users indicate damage to the dopaminergic and serotonergic systems, marked by decreases in the dopamine (DA) and serotonin (5-HT) transporters, DAT (McCann et al., 1998; Volkow et al., 2001a,b) and SERT (Sekine et al., 2006), which can last up to 3 years after the end of Meth use. PET and MRI observations in abstinent Meth users are substantiated by studies of human postmortem brain tissue and rodent studies. Postmortem studies of brains from chronic Meth users as well as rodents exposed to Meth, illustrate decreases in DA, tyrosine hydroxylase (TH), tryptophan hydroxylase (TPH), vesicular monoamine transporter -2 (VMAT-2), DAT and SERT, in areas highly innervated by DA and 5-HT terminals (Hotchkiss and Gibb, 1980; Ricaurte et al., 1980; Wagner et al., 1980; Wilson et al., 1996; Cass and Manning, 1999; Moszczynska et al., 2004; Eyerman and Yamamoto, 2007; Kitamura et al., 2007; Kish et al., 2009).

While the traditional belief is that Meth selectively damages DA and 5-HT axon terminals, more recent studies suggest that Meth produces more widespread damage. Meth users have decreases in the neuronal marker, N-acetylaspartate (Ernst et al., 2000; Chang et al., 2005), which is a more general indication of injury. In addition, rodent studies have shown that Meth produces damage to cell bodies evidenced by increased caspase-mediated proteolysis and TUNEL staining in the striatum and hippocampus (Schmued and Bowyer, 1997; Deng et al., 2001; Warren et al., 2005). Moreover, a study by Zhu et al. (2006) observed that parvalbumin-positive γ-aminobutyric acid (GABA) interneurons and cholinergic interneurons in the striatum undergo apoptosis after a single administration of 30 mg/kg Meth to mice. Meth binge treatment also decreases the number of glutamatergic neurons in the somatosensory cortex (Pu et al., 1996). Therefore, the toxic effects of Meth extend beyond dopaminergic and serotonergic terminals but the mechanisms mediating those effects remain to be determined.

## Mechanisms of monoamine damage

Many studies have investigated the mechanisms responsible for monoamine neurotransmitter damage caused by Meth. Those mechanisms include hyperthermia, neurotransmitter release, oxidative stress, mitochondrial dysfunction and inflammation.

Hyperthermia is an acute effect of Meth and appears necessary but is not sufficient to cause monoaminergic damage (Bowyer et al., 1992, 1994; Xie et al., 2000). This is evidenced by the fact that Meth-induced monoaminergic terminal damage is not observed in rodents that are placed in a setting with low ambient temperature and do not experience Meth-induced hyperthermia (Bowyer et al., 1992, 1994). Furthermore, pharmacological interventions that attenuate hyperthermia also attenuate Meth-induced monoaminergic damage. For example, antagonism of NMDA receptors using MK-801 or D1 or D2 dopaminergic receptors using SCH23390 and haloperidol, respectively, prevents Meth-induced hyperthermia and toxicity (Bowyer et al., 1994; Albers and Sonsalla, 1995). In contrast, several studies suggest that hyperthermia is not the sole mechanism since pharmacological interventions that do not alter hyperthermia will attenuate Meth toxicity (Itzhak et al., 2000; Ladenheim et al., 2000; Callahan et al., 2001; Sanchez et al., 2003).

Neurotransmitter release is a well-known acute effect of Meth. Meth increases extracellular concentrations of DA and glutamate (Raiteri et al., 1979; Mark et al., 2004). A consequence of these increases in cytosolic DA and extracellular glutamate is the generation of reactive oxygen species (ROS) and reactive nitrogen species (RNS). MAO metabolism of DA to DOPAC as well as autoxidation of DA results in the formation of ROS (Graham et al., 1978; Spina and Cohen, 1989; Hastings et al., 1996; Alper et al., 1999; Lavoie and Hastings, 1999). On the other hand, increased extracellular glutamate activates its calcium permeable receptors and results in the activation of nitric oxide synthase (NOS) (Dawson et al., 1991) and subsequent reactive nitrogen species (RNS) (Obata, 2002).

The role of ROS and RNS in Meth-induced monoaminergic terminal damage is well-established. Antioxidants such as N-acetyl-L-cysteine, ascorbic acid and vitamin E and NOS inhibitors attenuate Meth-induced striatal DA and 5-HT depletions (Wagner et al., 1985; De Vito and Wagner, 1989; Abekawa et al., 1996; Imam et al., 2001a; Fukami et al., 2004; Eyerman and Yamamoto, 2007). In addition, administration of Meth to mice overexpressing copper/zinc superoxide dismutase (Cu/ZnSOD) or manganese superoxide dismutase (MnSOD), enzymes responsible for eliminating superoxide radicals, or neuronal nitric oxide synthase (nNOS) knockout mice does not result in striatal damage (Cadet et al., 1994a,b; Hirata et al., 1995; Itzhak et al., 1998; Maragos et al., 2000; Imam et al., 2001b). In contrast, an SOD inhibitor enhances Meth-induced DA and 5-HT depletions in the rat striatum (De Vito and Wagner, 1989). Collectively, these data support the role of oxidative stress in Meth-induced neurotoxicity.

Excess extracellular glutamate can damage monoamine terminals independent of nNOS. Upon activation of its calcium permeable NMDA or AMPA receptors, glutamate results in an increase in intracellular calcium, which in turn activates calcium dependent enzymes and ultimately leads to cellular damage. In fact, Meth administration results in calpain-mediated spectrin proteolysis, which is associated with damage to striatal DA terminals (Staszewski and Yamamoto, 2006). In addition, it has been shown that inhibition of glutamate release from corticostriatal pathways or blockade of glutamate receptors prevents Meth-induced damage to striatal DA terminals (Sonsalla et al., 1991; Stephans and Yamamoto, 1994; Battaglia et al., 2002; Mark et al., 2004). Therefore, multiple studies support the role of excitotoxicity in Meth-induced monoaminergic terminal damage.

More recently, it was observed that Meth damages the liver, which can release factors known to mediate brain damage as in the case of hepatic encephalopathy. Binge Meth administration results in liver damage and consequent increases in circulating ammonia (Halpin and Yamamoto, 2012). Brain concentrations of ammonia are also elevated after Meth exposure and contribute to Meth-induced increases in extracellular glutamate (Halpin and Yamamoto, 2012; Halpin et al., 2014). Intrastriatal injection of the combination of Meth and ammonia produce decreases in monoamine tissue content that are prevented by the glutamate receptor AMPA antagonist, GYKI 52466 (Halpin and Yamamoto, 2012). In addition, treatment with an ammonia scavenger, lactulose, that is limited to the gut and does not enter the brain, attenuates Meth-induced monoamine depletions (Halpin and Yamamoto, 2012). These data together provide evidence that Meth not only damages the brain, but also damages peripheral organs, and the resulting increases in circulating ammonia contributes to Meth-induced monoaminergic damage in a glutamate dependent manner.

Mitochondrial dysfunction or bioenergetic stress is another mechanism by which Meth causes toxicity. Meth results in a depletion of ATP (Chan et al., 1994) and inhibits complex II of the electron transport chain (Brown et al., 2005) whereas treatment with the mitochondrial complex substrates, decylubiquinone or nicotinamide, after Meth treatment, attenuates Meth-induced striatal DA depletions (Stephans et al., 1998). Thus, a bioenergetic compromise and mitochondrial dysfunction play a role in Meth-induced monoaminergic terminal damage.

Less clear is the direct role of inflammation in Meth-induced monoaminergic terminal damage. Meth-induced monoaminergic damage is associated with neuroinflammation in humans and rodents. Recent PET scans of human Meth users indicate prominent microglial activation (Chang et al., 2002; Sekine et al., 2008) and chronic Meth users present with increased plasma cytokines and chemokines (Loftis et al., 2011). These observations are substantiated by preclinical studies that also observe glial activation (Escubedo et al., 1998; Lavoie et al., 2004; Thomas et al., 2004a,c) and increases in inflammatory mediators (Yamaguchi et al., 1991; Flora et al., 2002; Thomas et al., 2004b; Goncalves et al., 2008; Loftis et al., 2011) in various brain regions, including the hippocampus and striatum after Meth administration. Microglial activation is closely associated with Meth-induced monoaminergic damage and lack of microglial activation is typically associated with lack of monoaminergic damage (Lavoie et al., 2004; Thomas et al., 2004a,c). In addition, indomethacin attenuates microglial activation and decreases in beta III tubulin expression produced by Meth but monoaminergic depletions were not investigated (Goncalves et al., 2010). In contrast, microglial activation may be a consequence of Meth-induced damage, not limited to monoaminergic damage. The role of individual cytokines is less clear. Meth-induced monoaminergic terminal damage is prevented in IL-6 knockout mice (Ladenheim et al., 2000), suggesting a harmful role for IL-6. In contrast, intrastriatal injections of TNF-α attenuate rather than enhance Meth-induced decreases in DA and tyrosine hydroxylase (Nakajima et al., 2004), suggesting a protective role for TNF-α. Furthermore, Meth-induced DA depletions are still observed in TNF-α and COX-2 knockout mice, when Meth-induced hyperthermia is maintained (Nakajima et al., 2004; Thomas and Kuhn, 2005). Therefore, there is a tenuous relationship between inflammation and monoaminergic damage produced by Meth and the role of neuroinflammation in Meth-induced monoaminergic terminal damage warrants further investigation.

In summary, there is clear evidence that the acute effects of Meth, including hyperthermia, oxidative stress, excitotoxicity and mitochondrial dysfunction play a role in Meth-induced monoamine terminal damage. Research has focused primarily on the dopaminergic and serotonergic systems because of the direct pharmacological actions of Meth at DAT and SERT. However, the mechanisms responsible for monoaminergic damage are not specific for only neurons and likely cause damage to other cells in the brain. One class of cells that can be damaged by these mechanisms is those that which comprise the BBB.

## Mechanisms of BBB damage

The BBB can be disrupted by similar mechanisms that are responsible for Meth-induced monoaminergic damage and parallel those that are known to cause BBB disruption in a variety of other neurological diseases.

Extreme changes in body temperature are associated with increased BBB permeability. Kiyatkin and Sharma (2009) reported that both hyperthermia and hypothermia alter BBB function. Brains of rats, warmed to reach temperatures of 38.5–42°C, showed increases in albumin, GFAP-positive cells, water content and sodium, potassium and chloride concentrations, suggestive of brain edema. Moreover, the extent of BBB disruption was correlated with body temperature. Microwave-induced brain hyperthermia also results in horseradish peroxidase extravasation into the brain when brain temperatures were heated and maintained at 44.3°C for 30 min and 42.5°C for 60 min (Moriyama et al., 1991). Similarly, brain temperatures measured at hypothermic temperatures of 32–33°C also produced BBB leakage but to a lesser extent than hyperthermia (Kiyatkin and Sharma, 2009) and did not result in increases in brain water or ion content. These data suggest that alterations in brain temperatures alone can increase permeability of the BBB.

The role of oxidative stress in BBB disruption has been well-characterized. Oxidative stress is associated with BBB disruption in a variety of neurodegenerative disorders that have concomitant BBB disruption. Moreover, attenuation of oxidative damage is associated with protection against BBB damage (Kamada et al., 2007; Sharma et al., 2007; Ji et al., 2010, 2012; Price et al., 2012; Zehendner et al., 2013). Cerebrovascular endothelial cells in culture are damaged by 4-hydroxynonenal (HNE) (Karlhuber et al., 1997) and oxidized low-density lipoprotein (LDL), both of which are prevented by the antioxidant, resveratrol (Lin et al., 2010). Additionally, the free radical scavenger, edaravone, prevents oxidative stress, decreases in the BBB structural tight junction protein occludin, and increased BBB permeability to sodium-fluorescein produced by tissue plasminogen activator (tPA) in rats (Lukic-Panin et al., 2010). Furthermore, transgenic mice overexpressing copper/zinc superoxide dismutase are resistant to ischemia-induced free radical production and Evans blue leakage (Kim et al., 2001). In contrast, decreased copper/zinc superoxide dismutase activity enhances ischemia-induced edema in mice (Kondo et al., 1997). Together these findings illustrate a prominent role for oxidative stress in BBB damage after a variety of insults.

Mitochondrial dysfunction produces ROS and is also associated with BBB disruption in the context of Parkinson's Disease (PD) and Huntington's Disease (HD). Intrastriatal injection of the mitochondrial toxin 3-nitropropionic acid (3-NP), used as a model of HD, produced extravasation of Evans blue into the brain parenchyma as early as 3 h after administration of 3-NP while Evans blue extravasation and increased brain water content persisted for as long as 3 days after intrastriatal administration of 3-NP (Sato et al., 1997).

Excess extracellular glutamate can also lead to ROS and RNS production and possibly BBB disruption. Activation of ionotropic calcium permeable glutamate receptors, such as NMDA and AMPA receptors, results in an increase in intracellular calcium and subsequent activation of caspases and calpains as well as production of nitric oxide and reactive oxygen species (Siman and Noszek, 1988; Izumi et al., 1992). Consequently, increases in extracellular glutamate can be toxic to brain endothelial cells since they express NMDA receptors (Krizbai et al., 1998) and respond with increases in calcium influx and oxidative stress (Sharp et al., 2005; Neuhaus et al., 2011). In addition, exposure to glutamate decreases expression of the brain endothelial cell transmembrane protein, occludin (Andras et al., 2007; Neuhaus et al., 2011). Furthermore, increases in BBB permeability have been observed in response to seizure activity produced by a variety of convulsive agents (Nitsch and Klatzo, 1983; Pont et al., 1995) but it is unclear whether BBB disruption is a cause or consequence of seizure activity (for review, see Oby and Janigro, 2006). Thus, while excess extracellular glutamate likely contributes to BBB disruption, further studies are required to determine the role of glutamate in BBB disruption.

Lastly, inflammation is associated with BBB disruption. For example, the quintessential neuroinflammatory disease, Multiple Sclerosis (MS), presents with BBB disruption (Kermode et al., 1990). More specifically, TNFα expression and permeability of the BBB to endogenous albumin is produced by ischemia and treatment with an antibody against TNFα, attenuated ischemia-induced BBB damage (Yang et al., 1999). Pro-inflammatory mediators, such as cyclooxygenase (COX) also contribute to BBB disruption. COX inhibitors prevent ischemia- and TBI-induced BBB disruption (Mohanty et al., 1980; Ting, 1990; Candelario-Jalil et al., 2007; Hakan et al., 2010). Furthermore, prostaglandins are products of COX activity and mediate BBB disruption in a stroke model through EP1 receptor (EP1R) activation (Fukumoto et al., 2010). While it is unknown how EP1R activation increases BBB permeability, it may be through the signal transduction pathways that activate kinases (Bos et al., 2004) and subsequent phosphorylation of TJs. In fact, Rho kinase (ROCK) activity is stimulated by the EP1R (Wolkowicz et al., 2002), phosphorylates occludin and claudin-5 in brain endothelial cells (Yamamoto et al., 2008) and results in the loss of integrity of TJs in HIV-encephalitis models (Persidsky et al., 2006). Overall, there is significant support for the role of inflammation in BBB disruption.

## Meth-induced BBB damage

More recently, the BBB has been identified as a target of Meth and a change in the BBB is thought to be a consequence of the acute effects of Meth, similar to Meth-induced monoamine terminal damage. The majority of evidence is provided by preclinical studies that illustrate the effects of Meth on BBB structural proteins, the tight junction proteins, as well as BBB function.

Bowyer and Ali (2006) were the first to identify an increase in BBB permeability to endogenous IgG, as early as 90 min after exposure of rats to a single high dose of Meth, but this effect seemed to recover by 3 days after Meth exposure. Increases in IgG were apparent in the hippocampus and amygdala but only in rats that became hyperthermic and had seizures. Lower doses of Meth also increase permeability to Evans blue and FITC-labeled albumin within 24 h of Meth exposure (Kiyatkin et al., 2007; Kousik et al., 2011). These early increases in BBB permeability may be due to hyperthermia since the extent of increases in Evans blue and water content in the brain directly correlate with brain temperature (Kiyatkin et al., 2007). Transient changes in BBB structures have also been noted after Meth exposure. A single high dose of Meth to mice decreased expression of BBB structural tight junction proteins including occludin, claudin-5, and ZO, 24 h after Meth exposure (Martins et al., 2011). The mechanism underlying these changes may be associated with an increase in activated matrix metalloprotease-9 (MMP-9), which also has been demonstrated to play a major role in inflammation and ischemia-reperfusion-induced disruption of tight junction proteins and increase in BBB permeability (Mun-Bryce and Rosenberg, 1998; Yang et al., 2007). It remains to be determined whether MMP-9 plays a causative role in Meth-induced BBB disruption, however, hyperthermia contributes to the acute BBB disruption observed after a single high dose of Meth.

The contribution of oxidative stress in Meth-induced BBB disruption has also been studied. Exposure of cultured brain microvascular endothelial cells to Meth exhibit decreased expression of occludin, claudin-5 and zona occludens (ZO) as well as decreased transendothelial electrical resistance (TEER), indicative of increased paracellular permeability (Mahajan et al., 2008; Ramirez et al., 2009). These alterations in endothelial structures and function are associated with increases in ROS such that the antioxidant, Trolox, reduced Meth-induced decreases in TEER in endothelial cells in culture as well as extravasation of sodium-fluorescein in mice, observed 2 days after exposure to Meth (Ramirez et al., 2009).

Cells other than brain microvascular endothelial cells are responsible for BBB integrity. Astrocytes extend their endfeet to contact endothelial cells and help to stabilize and maintain BBB function (for review, see Abbott, 2000). One protein on astrocytes, β-dystroglycan, crosslinks astrocytic endfeet to endothelial cells and helps maintain a hydrophobic barrier and regulates aquaporin 4 (AQP4) localization. Thus, decreased expression of β-dystroglycan expression or truncation of its full form may prevent the localization of AQP4 to astrocytic endfeet and compromise the ability of AQP4 to regulate water clearance in the brain parenchyma (Papadopoulos et al., 2004). In fact, binge Meth exposure results in increased truncation of β-dystroglycan and increases in brain water content at 24 h (Northrop and Yamamoto, 2012). In addition, Meth acutely damages epithelial cells of the choroid plexus (Sharma and Kiyatkin, 2009), a structure involved in regulating water exchange between the brain and blood, which could also contribute to Meth-induced brain edema.

Another cell type that interacts with endothelial cells and helps to maintain proper BBB function are pericytes. Pericytes maintain proper BBB function including induction of tight junction and basement membrane formation, support of astrocytes in end-foot distribution and polarization and regulation of blood flow. Thus, an impairment of pericytes could dysregulate BBB function (for review, see Winkler et al., 2011). Pericytes can be damaged via mechanisms similar to those responsible for Meth-induced disruption of brain endothelial cells. For example, oxidative damage to pericytes in response to hyperglycemia and mitochondrial stress cause cell damage that can be prevented by antioxidant treatment (Price et al., 2012; May et al., 2014; Okada et al., 2014). Since pericytes express functional NMDA glutamate receptors (Montiel-Eulefi et al., 2012), they can be activated by Meth-induced glutamate release and in turn, lead to excitotoxicity. In addition, activation of glutamate receptors produces RNS and s-nitrosylation activates MMP-2 and MMP-9, two proteases known to disrupt tight junction proteins (Gu et al., 2002). Furthermore, inflammation may mediate the effects of Meth on pericytes as LPS infection can dissociate pericytes from endothelial cells and the basement membrane and increase BBB permeability (Nishioku et al., 2009).

Meth-induced peripheral organ damage, such as liver, muscle and kidney, can also lead to BBB disruption. Peripheral organ damage can lead to increases in circulating and brain ammonia concentrations through increases in brain glutamate (Chan et al., 2000; Gorg et al., 2010; Halpin et al., 2014). Ammonia can also induce oxidative stress via superoxide and nitric oxide production as well as through decreases in antioxidant enzymes (Kosenko et al., 1997a,b). Consequently, ammonia may result in oxidative damage of endothelial cells or activation of MMPs. In addition, excess ammonia can result in neuroinflammation marked by increases in microglial and astrocytic activation, pro-inflammatory cytokines and prostaglandin E2 (PGE2) (Rodrigo et al., 2010; Bemeur and Butterworth, 2012). Thus, ammonia may also be a contributing factor to Meth-induced BBB damage via oxidative, excitotoxic and/or inflammatory dependent manners.

## Meth comorbidities

It is important to note that Meth use, similar to other drugs of abuse, does not occur in isolation. Comorbidities associated with Meth, including stress disorders, use of other drugs and HIV infection, may potentiate the effects of Meth on the BBB.

### Stress

Stress is highly comorbid with drug use. Stressors increase ROS (Mcintosh and Sapolsky, 1996; Leza et al., 1998; Madrigal et al., 2001), inflammatory mediators (Minami et al., 1991; Garcia-Bueno et al., 2008), mitochondrial dysfunction (Madrigal et al., 2001) and extracellular glutamate (Moghaddam, 1993). As mentioned previously, these mechanisms contribute to Meth-induced BBB disruption in response to a variety of insults. Thus, it is likely that stress could exacerbate Meth-induced BBB disruption by potentiating Meth-induced increases in ROS, inflammatory mediators, mitochondrial dysfunction, and extracellular glutamate.

In fact, serial exposure to 10 days of chronic unpredictable stress (CUS) and Meth produce an enhanced damage of the BBB, compared to stress or Meth alone at 24 h after Meth exposure. This damage was evidenced by decreases in the tight junction proteins, occludin and claudin-5, truncation of the astrocytic endfeet protein β-dystroglycan, as well as increases in BBB permeability to water and 10,000 Da FITC-dextran (Northrop and Yamamoto, 2012). Furthermore, the combination of stress and Meth but not Meth alone, prolonged BBB disruption in a manner that was paralleled by an increase in inflammatory markers, GFAP and COX-2, up to 7 days after exposure to stress and Meth (Northrop and Yamamoto, 2012). In addition, the non-selective COX inhibitor, ketoprofen, prevented the disruption in BBB structure and function observed at 7 days, when ketoprofen was administered after, but not during exposure to stress and Meth (Northrop and Yamamoto, 2012). This illustrates that the combination of stress and Meth produces a delayed increase in neuroinflammation that is responsible for the prolonged breach in the BBB. Therefore, these findings illustrate that stress enhances Meth-induced early BBB disruption and prolongs the opening of the BBB, as compared to Meth alone in a manner that is dependent on COX activity. Other mechanisms by which stress can enhance Meth-induced BBB damage, such as oxidative stress, mitochondrial dysfunction and glutamate remain to be examined.

### Ethanol

Ethanol is one drug that is frequently abused in combination with Meth. Ethanol can directly damage brain microvascular endothelial cells (BMVECs) in culture. Exposure to ethanol produces activation of myosin light chain kinase and consequent phosphorylation and degradation of tight junction proteins, occludin, claudin-5 and ZO-1. Furthermore, a decrease in barrier function evidenced by a decrease in TEER and an increase in monocyte migration are observed in BMVECs exposed to ethanol. Moreover, the effects of ethanol on tight junction proteins and barrier function were associated with increases in ROS, resulting from ethanol metabolism, and increased calcium concentrations and were prevented by antioxidant treatment or an inhibitor of the intracellular calcium modulator, inositol 1,4,5-triphosphate receptor (IP3R) (Haorah et al., 2005, 2007). The effects of ethanol on the BBB in rodents however are less clear. Chronic treatment of rats with 20% ethanol in drinking water for 12 months produces BBB disruption as evidenced by increased BBB permeability to endogenous IgG in the cortex (Ehrlich and Humpel, 2012). Similarly, 2 weeks of a 5% liquid alcohol diet increased BBB permeability to radioactively labeled leptin (Pan et al., 2008). In contrast, 7.5% ethanol in drinking water for 6 months had no effect on permeability of the BBB to sucrose (Phillips, 1981) nor did acute ethanol intoxication affect BBB permeability (Hemmingsen et al., 1980; Pan et al., 2008). Moreover, some studies suggest that acute ethanol may even protect against BBB disruption resulting in stroke models (Zeng et al., 2012; Peng et al., 2013), but others suggest a detrimental effect of ethanol in HIV Tat and gp120-induced endothelial cell death and BBB permeability (Acheampong et al., 2002; Shiu et al., 2007). Thus, ethanol alone can have protective or detrimental effects on BBB function depending on dose and length of exposure. In combination with Meth, however, ethanol could potentiate Meth-induced BBB disruption through its pro-inflammatory (Le Moine et al., 1995; Mathurin et al., 2000) and pro-oxidant (Haorah et al., 2007) effects.

### HIV infection

Meth use is also highly comorbid with HIV infection and the combination of HIV infection and Meth can enhance BBB disruption. The BBB is affected by HIV infection and is also involved in the progression of disease (for review, see Banks et al., 2006). While HIV-induced increases in BBB permeability may not be the sole reason for immune cells and virus infiltration into the brain, HIV proteins act on BMECs and result in increased neuroinflammation. Therefore, HIV infection could exacerbate Meth-induced BBB damage.

The HIV proteins, Tat and gp120, alone or in combination, cause BBB disruption via oxidative and inflammatory mechanisms (Toborek et al., 2003; Price et al., 2005). Decreases in tight junction proteins, claudin-1, claudin-5, and ZO-2 in BMVECs as well as cytotoxicity of BMVECs were observed after treatment with HIV Tat (Andras et al., 2003; Khan et al., 2003; Kim et al., 2003). Furthermore, acute administration of gp-120 or cellular expression of gp-120 caused the extravasation of Evans blue into the brain and decreased the tight junction protein, claudin-5. These alterations in BBB were associated with increased expression of MMP-2 and MMP-9. Moreover, when MMP expression was inhibited via antioxidants or an NMDA receptor antagonist, the gp-120-induced BBB disruption was prevented (Louboutin et al., 2010). Therefore, HIV infection may exacerbate Meth-induced BBB disruption via oxidative, inflammatory or glutamate dependent mechanisms.

In fact, administration of the HIV proteins Tat and gp120 in combination with Meth to mice decreased tight junction protein expression and increased BBB permeability to sodium-fluorescein. These changes were associated with decreases in glutathione and increased oxidation of lipids and proteins that were attenuated by treatment with the antioxidant, N-acetylcysteine amide (NACA) (Banerjee et al., 2010). This further supports the interaction of HIV infection and Meth at the BBB as well as role for oxidative stress in HIV and Meth-induced BBB disruption. However, more investigation is necessary for a clear understanding of the interaction of HIV infection and Meth in humans as well as the role of inflammation and glutamate in HIV and Meth-induced BBB disruption.

## Consequences of meth-induced BBB disruption

The opening of the BBB can have broad and significant consequences, particularly with regard to Meth use. These consequences may manifest as enhanced vulnerability to other disease states that are co-morbid with Meth use such as stress, HIV infection (Swan, 1997) and Hepatitis C. In fact, Meth users have higher Hepatitis C and HIV viral titers in their brain as compared with non-Meth users (Letendre et al., 2005, 2007; Nath, 2010).

Meth abuse is also associated with other infections. Meth abuse produces what has been informally termed “Meth mouth.” “Meth mouth” is characterized by extensive tooth decay, bruxism, and dry mouth (Rhodus and Little, 2008), all of which can lead to bacterial infections and periodontal gum disease. A primary culprit of periodontal disease is *Porphyromonas gingivalis (P. gingivalis*), a bacterium also linked to rheumatoid arthritis and atherosclerosis (Iwai, 2009; Berthelot and Le Goff, 2010). Few studies have examined its appearance and action in the brain, although there is evidence that *P. gingivalis* can activate microglial cells in culture (Shapira et al., 2002). Therefore, the brains of Meth users could be extremely vulnerable to infection by HIV, Hepatitis C and *P. gingivalis*, which can lead to enhanced inflammatory responses and further brain damage.

More generally, Meth-induced BBB disruption may be the cause of the cognitive decline and depression seen in Meth users. BBB disruption is a pathological symptom of Alzheimer's disease and BBB disruption is thought to play a role in cognitive decline and dementia (for review, see Van De Haar et al., 2014); however, the exact mechanisms are unknown. BBB disruption also leads to increases in infiltration of immune cells and inflammatory mediators, which could affect normal brain physiology and function. For example, neuroinflammation is thought to precipitate depression (for review, see Najjar et al., 2013) and may underlie the depression associated with long-term use of Meth.

In contrast to the negative consequences of BBB disruption, an opening of the BBB may facilitate the entry of therapeutics that normally cannot enter the brain such as those used to treat some neurodegenerative disorders or brain cancers. Thus, basic knowledge about how Meth opens the BBB and the mechanisms that underlie its acute and long-term effects may provide clues as to how transient and persistent changes in the BBB can be differentiated by other means than Meth exposure to facilitate acute entry of therapeutic drugs into the brain.

## Conclusion

Meth results in disruption of BBB structure and function and the mechanisms that contribute to BBB disruption are similar to those that are responsible for monoaminergic terminal damage after Meth. However, more studies are needed to assign a causative role for inflammation, glutamate and ammonia in Meth-induced BBB disruption. Furthermore, comorbidities of Meth including stress, poly-drug use and HIV infection could exacerbate the damaging effects of Meth on the BBB. These findings and possibilities highlight the urgency for new research to identify the precise mechanisms that lead to the development of therapeutics to treat the far-reaching and significant consequences of Meth-induced BBB disruption.

### Conflict of interest statement

The authors declare that the research was conducted in the absence of any commercial or financial relationships that could be construed as a potential conflict of interest.
